# Supplementary material for: GABRP promotes CD44s-mediated gemcitabine resistance in pancreatic cancer
Source: PeerJ. 2022 Jul 11;10:e12728. doi: 10.7717/peerj.12728 (PMC9281597; doi:10.7717/peerj.12728)
Supplement: Supplemental Information 1 [file peerj-10-12728-s001.docx]

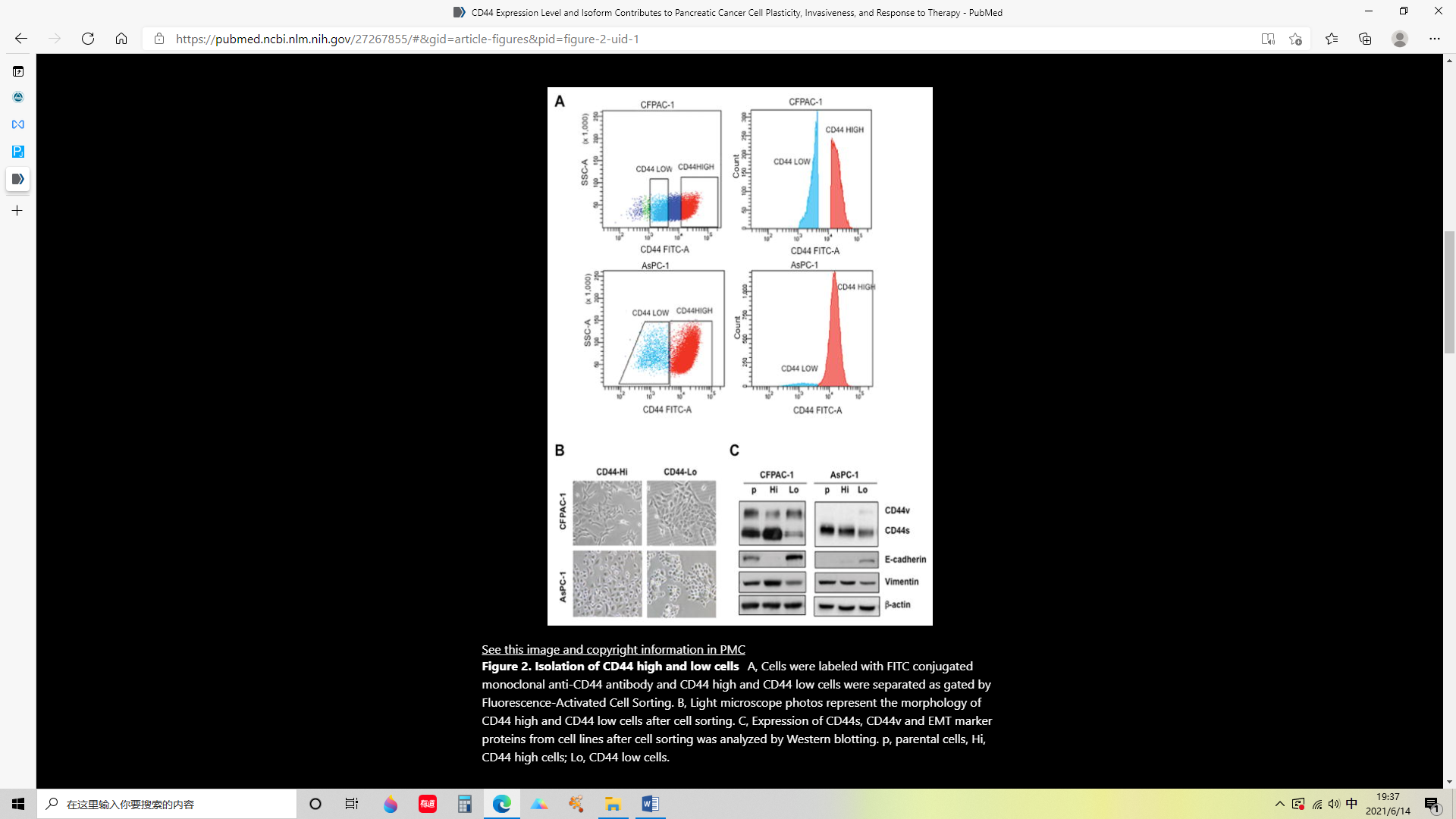


Zhao S, Chen C, Chang K, Karnad A, Jagirdar J, Kumar AP, Freeman JW. CD44 Expression Level and Isoform Contributes to Pancreatic Cancer Cell Plasticity, Invasiveness, and Response to Therapy. Clin Cancer Res. 2016 Nov 15;22(22):5592-5604. doi: 10.1158/1078-0432.CCR-15-3115. Epub 2016 Jun 7. PMID: 27267855; PMCID: PMC5143222.

I published this paper as co-first author in 2016. Flowcytometry separated out CFPAC-1-CD44 High and Low expressing cells, then I seeded a few number of cells in 10cm petri dish to single clone out cells.


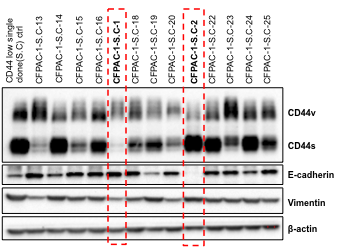


In this context, figure 3C, CF-cl.21-ctrl is CD44 High cells (CFPAC-1-S.C.2), we knockdown CD44 from CFPAC-1-S.C.2. In the same way, we selected out 2 knockdown clones. (Unpublished data, I will submit this figure in another paper, hope you will understand, thank you so much.)


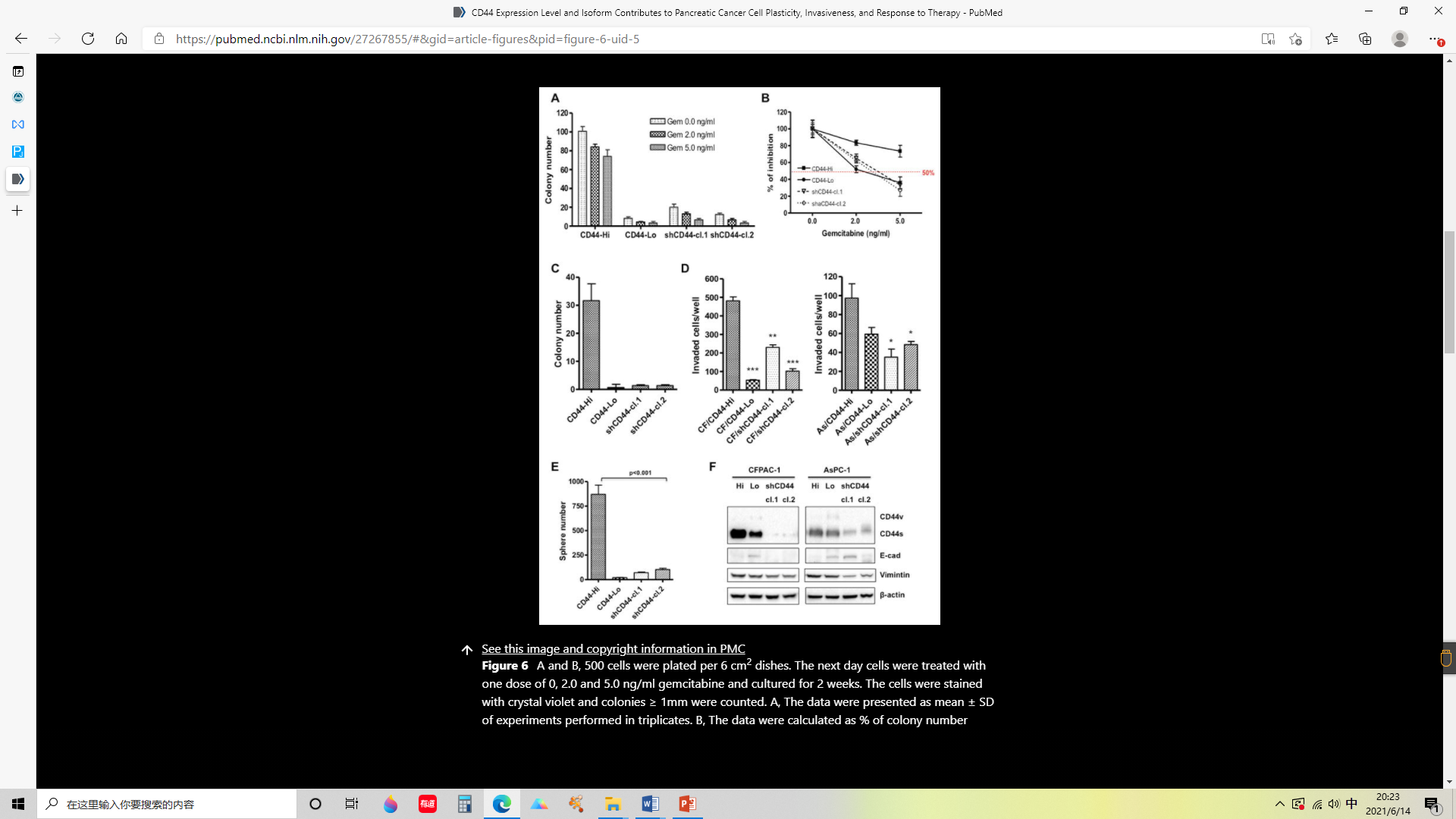


Zhao S, Chen C, Chang K, Karnad A, Jagirdar J, Kumar AP, Freeman JW. CD44 Expression Level and Isoform Contributes to Pancreatic Cancer Cell Plasticity, Invasiveness, and Response to Therapy. Clin Cancer Res. 2016 Nov 15;22(22):5592-5604. doi: 10.1158/1078-0432.CCR-15-3115. Epub 2016 Jun 7. PMID: 27267855; PMCID: PMC5143222.

Two shCD44 cl.1 and cl.2 were already published in my previous publication.


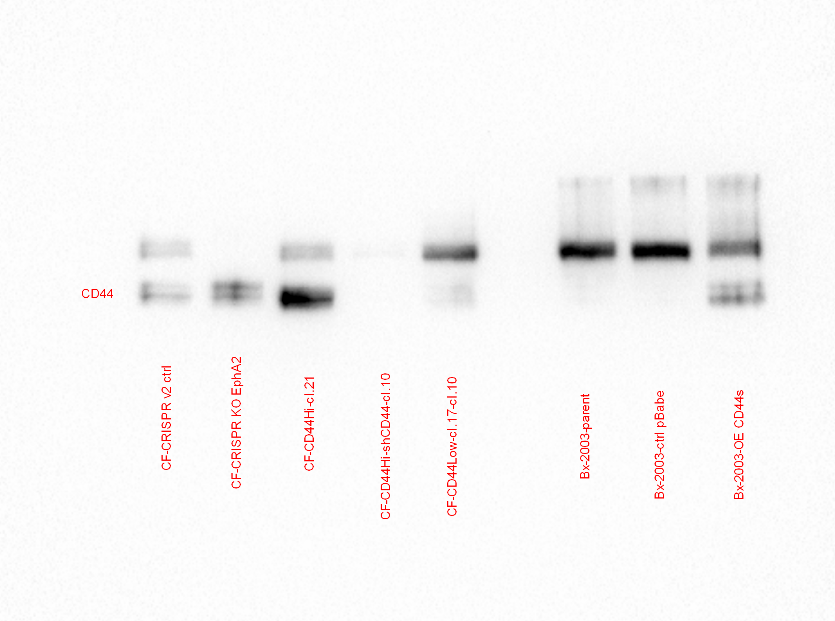


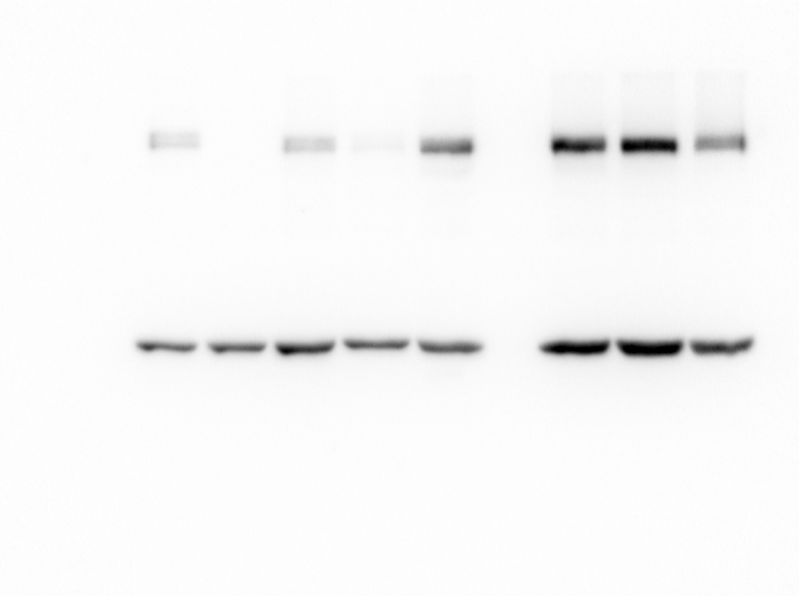


The third and fourth lane represented Figure 3b original data. Upper figure is CD44 and lower figure is actin.
